# Vascular Age, Metabolic Panel, Cardiovascular Risk and Inflammaging in Patients With Rheumatoid Arthritis Compared With Patients With Osteoarthritis

**DOI:** 10.3389/fcvm.2022.894577

**Published:** 2022-07-05

**Authors:** Gabriel-Santiago Rodríguez-Vargas, Pedro Santos-Moreno, Jaime-Andrés Rubio-Rubio, Paula-Katherine Bautista-Niño, Darío Echeverri, Luz-Dary Gutiérrez-Castañeda, Fabio Sierra-Matamoros, Stephania Navarrete, Anggie Aparicio, Luis Saenz, Adriana Rojas-Villarraga

**Affiliations:** ^1^Research Institute, Fundación Universitaria de Ciencias de la Salud-FUCS, Bogotá, Colombia; ^2^Rheumatology, Biomab - Center for Rheumatoid Arthritis, Bogotá, Colombia; ^3^Research Laboratory, Fundación Cardiovascular de Colombia (FCV), Bucaramanga, Colombia; ^4^Cardiovascular Prevention Program, Fundación Cardioinfantil-Instituto de Cardiología, Bogotá, Colombia; ^5^Basic Sciences Laboratory, Fundación Universitaria de Ciencias de la Salud-FUCS, Bogotá, Colombia; ^6^Epidemiology, Fundación Universitaria de Ciencias de la Salud (FUCS), Bogotá, Colombia

**Keywords:** rheumatoid arthritis, osteoarthritis, lipid profile, pulse wave velocity, inflammaging, vascular age

## Abstract

**Introduction:**

The risk of cardiovascular disease (CVD) in patients with rheumatoid arthritis (RA) is 1.5–2 times higher than the general population. The fundamental risk factor for CVD is age, related to alterations at the arterial level. The aim of the study was to compare vascular age (VA) in RA patients under a strict treat-to-target (T2T) strategy with Osteoarthritis (OA) patients without strict follow up and to assess the influence of inflammaging (chronic, sterile, low-grade inflammation related to aging) and metabolic markers on VA.

**Materials and Methods:**

This was an analytical cross-sectional study. Patients with RA (under a strict a T2T strategy) and OA patients without strict clinical follow-up were included. Patients with a history of uncontrolled hypertension, CVD, and/or current smoking were excluded. Sociodemographic, physical activity, and toxic exposure data were obtained. Waist-hip ratio and body mass index (BMI) were measured. DAS-28 (RA) and inflammatory markers, lipid profile, and glycaemia were analyzed. Pulse wave velocity (PWV) was measured (oscillometric method, Arteriograph-TensioMed®). VA was calculated based on PWV. Eleven components of inflammaging [six interleukins, three metalloproteinases (MMP), and two tissue inhibitors of metalloproteinases (TIMP)] were evaluated (Luminex® system). Univariate and bivariate analyzes (Mann Whitney U and chi-square) and correlations (Spearmans Rho) were done to compare the two groups.

**Results:**

A total of 106 patients (74% women) were included, 52/RA and 54/OA. The mean age was 57 (Interquartile range - IQR 9 years). The BMI, waist circumference, and weight were higher in patients with OA (*p* < 0.001). RA patients had low disease activity (DAS-28-CRP). There were no differences in VA, inflammaging nor in PWV between the two groups. VA had a positive, but weak correlation, with age and LDL. In group of RA, VA was higher in those who did not receive methotrexate (*p* = 0.013). LDL levels correlated with MMP1, TIMP1, and TIMP2.

**Conclusions:**

When comparing RA patients with low levels of disease activity with OA patients with poor metabolic control, there are no differences in VA. Furthermore, methotrexate also influences VA in RA patients. This shows that implemented therapies may have an impact on not only the inflammatory state of the joint but also CVD risk.

## Introduction

Rheumatoid arthritis (RA) is a chronic, inflammatory, autoimmune, and systemic disease which is characterized by inflammation in the synovial membrane that causes joints swelling, pain, and stiffness and could generate other extra-articular manifestations (1, 2). Within the epidemiological profile of Colombian patients with RA, the most frequent comorbidities observed are arterial hypertension - 31.15%, osteoporosis - 19.46%, and polyautoimmunity with Sjögren's syndrome - 10.4% (3, 4). The DMARDs used the most are methotrexate and chloroquine, and Anti-TNF as biological therapy. Treatment with leflunomide or rituximab has been shown to be associated with a lower probability of achieving remission (5). Also, in Colombia, the ethnic distribution of patients with RA is mainly represented by the mestizo population at 60.9%. This is similar to the rest of the Latin American population and corresponds to approximately 667 million inhabitants who also share other characteristics such as socioeconomic, environmental factors, and ancestry (6). Therefore, this population could represent Latin America population.

The risk of cardiovascular mortality for patients with RA is up to 50% higher compared to the general population, and the risk of myocardial infarction is double (7). In 2016, the European League Against Rheumatism (EULAR) recommended that a 1.5 multiplication factor be used to adapt the cardiovascular disease (CVD) risk prediction models to patients with RA (8). Some studies show that if the disease activity is reduced, mortality could be reduced by up to 20%. There may also be a risk reduction of up to a maximum of 50% from high disease activity to remission (7). This is achieved in particular when a treat-to-target strategy (T2T) that consists of strict disease monitoring is adopted in order to bring about disease remission and control of the inflammatory state under a multidisciplinary team approach (9).

The role of long-standing inflammation as a primary mechanism of chronic disease and aging is being actively investigated. The term inflammaging was coined to described the sterile, chronic, low-grade inflammatory process which develops with age, and brings susceptibility to various age-related illnesses (10, 11). Thus, patients suffering from autoimmune diseases such as RA with high levels of disease activity are in a chronic proinflammatory state derived from immune auto reactivity, which leads to accelerated aging and affects multiple systems including the cardiovascular system (7, 10).

Pulse wave velocity (PWV) is currently considered the gold standard for non-invasive measurement of arterial stiffness, and this parameter makes it possible to evaluate cardiovascular risk for major endpoints (12, 13). The value that determines arterial stiffness has been extensively studied. In 2010, the European society of cardiology created reference values for PWV based on age and blood pressure (BP) level using the reference values established by the Arterial Stiffness Collaboration Study Group (12). These reference values become important with respect to age because vascular age (VA) can be calculated based on the PWV value (12).

In fact, there are studies evaluating the PWV in RA but not many that assess VA, and few or none that analyze VA based on PWV in RA patients (14–19). For instance, Mong et al. used the coronary calcium score (CCS) as a non-invasive method to calculate arterial age and cardiovascular risk stratification. They found a significant increase and faster vascular aging in RA patients compared to the control group (20).

There are no studies that compare the state of VA, arterial stiffness, metabolic markers, and inflammaging in patients with RA and Osteoarthritis (OA). OA is considered a degenerative or mechanical disease rather than an autoimmune disease and, in various settings, it has been considered to have a lower burden of cardiovascular risk (21, 22) when compared to systemic diseases such as RA. In addition, patients of both entities are frequently seen in the rheumatology setting. However, in various scientific scenarios these two pathologies are compared, not only because of their frequency in the field of rheumatology, but also because of the burden of disease that they produce (23). Some common points within their pathophysiology (24, 25), although they are of a different nature, the cardiovascular risk inherent to some medications that are commonly used in the treatment of the two diseases (26–29) and the possible cardiovascular risk that both diseases could have, being in theory, lower in OA (for instance, metabolic risk) (30–32).

The hypothesis of the present study was that CVD risk, measured with PWV and VA does not increase in RA patients when the inflammatory activity of disease is under very good control in comparison to osteoarthritis patients. Therefore, the focus of the study was to compare VA and arterial stiffness (PWV) in two group of patients, one group with RA under a strict T2T strategy and the other group with OA (a complex multifaceted disease associated with aging and inflammation but with lower cardiovascular risk compared to RA) (33). The association of inflammaging and metabolic and clinical markers with VA was also assessed.

## Materials and Methods

### Study Design and Sample Size

This was an Analytical cross-sectional study carried out on patients with RA and a reference population with OA between March and September of 2021.

The sample size calculation was based on studies in which the PWV values and differences (to assess vascular stiffness) between patients with RA and controls without RA were estimated (14, 15, 17). A power of 80% and a type I error of 0.05 were assumed. The calculation yielded 50 patients with RA and 50 patients with OA for a total of 100 patients.

### Study Population

The study subjects were patients with RA who were treated at a specialized Rheumatology center in Bogota, Colombia under a T2T strategy with strict control of the disease. They came monthly for regular checkups by a multidisciplinary team. Also, patients at the same Rheumatology center with OA but no history of RA or autoimmune diseases were included. This study, including procurement of informed consent was approved by the ethic committee for research on human beings (CEISH) by hospital de San José HSJ-FUCS (Identifier 0173-2019).

### Inclusion and Exclusion Criteria

#### Inclusion Criteria for Patients With Rheumatoid Arthritis and Osteoarthritis

Patients between 40 and 70 years old, with a confirmed RA diagnosis (International Classification of Diseases: M069, M059, M060) were classified as having RA if they fulfilled the American College of Rheumatology classification criteria for RA (34). Patients with OA, treated at the same center, between 40 and 70 years old, were under a non-strict follow up program (one or two visits per year).

#### Exclusion Criteria for Patients in Both Groups

Patients with a known diagnosis of juvenile arthritis based on international criteria before the age of 16 (35); patients with a known history of CVD or cerebrovascular disease or other types of peripheral vascular disease, coronary heart disease, transient ischemic attack, angina, use of nitroglycerin, history of heart failure, atrial fibrillation, or other arrhythmia diagnosed by a physician; patients who have undergone a surgical procedure related to CVD like angioplasty, valve replacement, coronary bypass, pacemaker, or defibrillator implantation or any surgery involving the heart or large arteries were not included. Also, patients with arterial hypertension (AH) as defined by the American heart association (36) were excluded with the exception of those patients who were under treatment and had reached the BP control target. Patients with high cardiac output like anemia with hemodynamic compromise and the presence of fever were also excluded.

Other exclusion criteria were: patients with obesity grade II [Body mass index (BMI): ≥ 35 kg/m^2^ – 39.9 kg/m^2^] or obesity grade III [BMI: ≥ 40 kg/m^2^] (37); type 2 diabetes mellitus, with poor glycemic control (38); patients with chronic kidney disease (39); patients with active treatment for cancer, or an active infectious process like SARS-CoV-2 infection; patients with liver cirrhosis (40); pregnancy; patients who consume stimulant drugs and alcohol (41) and active smokers were excluded (42). We excluded those variables because they have an influence on PWV results and could be confounding variables (43–71).

### Recruitment Strategy

Patients were randomly selected by sampling and were chosen from the universe of patients who go to the specialized rheumatology center. Patients who met the inclusion criteria based on clinical records and patient information were contacted by phone. Patients who agreed to participate were given an appointment and informed consent was obtained. Patients were evaluated in groups of 10 for blood samples and clinimetric measurements. No more than a month later, they underwent a non-invasive vascular test.

All patients included in the study had to undergo a nasal antigen test for coronavirus disease 2019 (COVID-19) (given the pandemic during the period when the study was done). Blood samples were taken to process metabolic markers, acute phase reactants, autoantibodies, and the inflammaging. Non-invasive vascular tests were measured with an Arteriograph® (Tensiomed, Budapest) at the La Cardio- Fundación Cardioinfantil of Bogotá using the established protocol.

### General and Sociodemographic Data

Electronic medical records were reviewed and patient interviews were done to obtain information about demographic characteristics such as gender, age, marital status, educational level, current occupational status, comorbidities, COVID-19 vaccine status, cardiovascular family history, familial autoimmunity, physical activity, and toxic exposure. All variables were corroborated by a patient doctor visit.

### Clinical Characteristics by Disease

Data on age at onset of symptoms, age at diagnosis, duration of disease, presence of erosivity, extra-articular manifestations, positivity for rheumatoid factor (RF) and/or anti-citrullinated protein antibodies (ACPA), presence of polyautoimmunity, and treatment were obtained on patients with RA. Pain visual analog scale (VAS) 0–10 cm and physician global assessment using VAS 0–10 scores were obtained for both groups. Disability was evaluated using the multi-dimensional health assessment questionnaire (MD-HAQ) (72), and the routine assessment of patient index data 3 (RAPID3) was calculated (73, 74). The Quality of life was assessed with the European Quality of Life-5-dimension-3- level instrument (EQ-5D-3L). For EQ-5D-3L calculations, an overall index score [that measures from the lowest (worst) to the highest (best) score] was calculated for each patient and time point. The time trade-off (TTO) valuation technique was used. The value sets from the Spanish population were chosen since none are currently available for Colombia. State of health was also measured on the vertical VAS (score 0 to 100), where higher scores equal better health status (75). Disease activity in RA patients was measured using the disease activity score with 28-joint counts (DAS-28) (76) [interpreted as high (DAS-28 > 5.1), moderate (DAS-28 ≤ 5.1–≥3.2), low (DAS-28 < 3.2–≥ 2.6), and remission (DAS-28 < 2.6)]. In OA patients, the presence of concomitant musculoskeletal diseases was evaluated based on the physical exam and clinical record. Information regarding treatment was obtained (physical therapy, evaluation by physical and rehabilitation medicine, and history of medications).

### Laboratory Variables

For blood sampling, the patient was informed of the need to fast 8 h prior to the procedure. A total volume of 12 cc (3 tubes of 4 cc each) was collected to detect biomarkers. The laboratories and technique that were applied were: Fasting plasma glucose (FPG), triglycerides (TG), total cholesterol (TC), high-density lipoprotein cholesterol (HDL-C), and low-density lipoprotein cholesterol (LDL-C) with the enzymatic method; rapid antigen test for COVID-19, C-reactive protein (CRP), and RF with the immunoturbidimetric method; erythrocyte sedimentation rate (ESR) with the Westergreen method, glycosylated hemoglobin A1c (HbA1c) with capillary electrophoresis; thyroid-stimulating hormone (TSH) with Chemiluminescence, ACPA with micro-ELISA; and Antinuclear antibody (ANA) with IFI on Hep-2 cells method.

Inflammaging analysis included: TNF-α, IFN-γ, IL-1β, IL-1 RA, IL-6, IL-10, metalloproteinases (MMP-1/2/9), and tissue inhibitors of metalloproteinases (TIMP 1 and 2). They were quantified using the HCYTOMAG-60K kit for classical cytokines, the HMMP2MAG-55K kit for metalloproteinases and the HTMP2MAG-54K kit for TIMP in a Milliplex® MAP platform following the manufacturer's instructions. Briefly, serum aliquots with no freeze-thaw cycles were completely thawed, mixed with a vortex, and centrifuged prior to processing. For additional dilutions, serum matrix was used. The night before processing, samples were incubated at 4°C with antibody-immobilized beads in 96-wells plates. The next day, the plate was washed twice with a buffer solution and incubated with secondary detection antibodies for 1 h, followed by incubation with Strepavidin-PE for 30 min. Once the second wash was done with the buffer solution, the sample was run in the Luminex® system. Standard curves were used to calculate the biomarker concentrations in each sample. All samples were run in duplicate. The Analyst 5.0® software was used for analysis.

### Cardiovascular Variables

Cardiovascular measurements using the Arteriograph TensioMed® included: BP with the appropriate and validated technique (36), diastolic BP (DBP), systolic BP (SBP), pulse pressure (PP), mean arterial pressure (MAP), central systolic BP (cSBP), and heart rate (HR). The Arteriograph technique was as follows: Patients were told to refrain from alcohol, coffee, tea, or beverages containing caffeine or cocoa for 12 h prior to the examination. Patients rested for 5 min supine in a temperature-controlled room. During the examination, patients were asked not to move, speak, or cough. For the measurement of the PWV, an additional requirement was to approximate the length of the aorta by measuring the distance (in cm) between pubic bone and suprasternal notch. Prior to the determination of the stiffness parameters, SBP and DBP were measured on the left and right upper arm and lower leg. The PWV measurement was taken with inflated BP cuffs on the right upper arm and the right lower leg to oscillometrically detect pulsatile pressure changes in the brachial and anterior tibial artery. The examination was divided into two parts. First, PWV brachial-ankle (PWVba) was determined by inflating both cuffs at the predetermined DBP level for 15 s. Afterwards, PWV aortic (PWVao) and aortic augmentation index (AIao) were obtained by applying a cuff pressure of 35 mmHg above the SBP on the upper arm. Then, the Vascular Explorer software analyzed all recorded pressure waves in each cycle and calculated the mean of the measured values. The values of the pressure wave that measured closest to the mean values was chosen for quantitative analysis. To calculate the PWVba, the Vascular Explorer software first imputes distances derived from patient height and utilizes the pulse transit time (77). According to the model applied by the Vascular Explorer for PWV measurement, the second pressure wave in the brachial artery pressure contour represented the wave reflection at the aortic bifurcation. The additional distance traveled by this reflected wave compared to the initial pressure wave in the brachial artery thus represented an approximation of two times the length of the aorta. By measuring the time difference between the arrival of both pressure waves in the brachial artery PWVao could be determined non-invasively. The carotid-femoral PWV (PWVcf) was derived from the PWVao and PWVba by applying an unpublished formula. Transfer functions that are also obtained from the Vascular Explorer software provided central pressure waves thus allowing the calculation of AIao via peripheral pressure waves. Finally, the PWVao, the PWVba, and the AIao were used to calculate a VA (VAPWV) depending on respective PWV and AI measurements: VA_PWVao_, VA_PWVba_, and VA_AIao_. VA was based on nomograms provided by McEniery et al. (78). Each measured value was assigned to an age at which it corresponds to the 50th percentile in the cohort named (77). In addition, waist circumference, hip circumference, waist-hip ratio, weight (kg), height (cm), and BMI (kg/m^2^) were measured. Physical activity, type of exercise (aerobic or anaerobic), the frequency of the exercise, and the minutes of exercise were obtained through a questionnaire given on the date of the appointment. Framingham risk score adjusted for Colombia (FRS) (79) was calculated in both groups. In addition, FRS was adapted according to EULAR 2016 recommendations by a 1.5 multiplication factor (8).

Also, two novel concepts regarding VA were applied to classify the patients: early vascular aging (EVA), which refers to premature arterial stiffness for age assessment and supernormal vascular aging (SUPERNOVA), which refers to the fact that regardless of risk factors and age, arteries did not lose their elasticity and correspond to the same or a younger VA than biological age (80).

### Statistical Plan and Data Sources

Two expert researchers collected all the information using the Research Electronic Data Capture (REDcap) platform [(https://www.project-redcap.org/ (accessed on 2021]). A CSV data file was imported into SPSS® v21 for statistical analysis.

Measurements of central tendency and dispersion were applied for quantitative variables and qualitative variables were described by means of absolute and relative frequencies. Bivariate analyses were done to compare the main sociodemographic, clinical, laboratory, inflammaging, and cardiovascular measurements between the two groups of patients.

VA differences between RA patient and OA patients were assessed by means of Mann Whitney U or t-Student tests depending on the distribution of the variable. Both the relationship between the VA variable and each biomarker of inflammaging, or clinical, laboratory or metabolic characteristics and the one between lipid panel and inflammaging components were evaluated for each group of participants, with and without RA, using Pearson's or Spearman's correlations, Chi2 test or Fisher's test (81).

Due to the fact that the Arteriograph® reports VA values greater than or equal to 60 years (not the exact VA) in some individuals, a multiple imputation process was done to obtain exact values. First, the Missing Values Analysis in the SPSS® package to describe the pattern of missing data was done. Second, the automatic function to scan the data for monotonicity was applied. Next, the standard linear regression model was chosen. Then, VA was set as an outcome variable and PWV was set as a predictor variable given that the remaining data regarding VA were calculated by the Arteriograph based on PWV. Finally, the multiple imputation was done and, as a result of the iterated process, a new data set was created for the imputed data. This data set contained imputed values in place of the reported VA values indicated as ≥60 Ys. The data set included five imputations carried out in sequence. Those 5 values were averaged, and the resulting single VA value was used in the primary analysis.

## Results

### Sociodemographic and Baseline Characteristics

A total of 106 patients were included (Table 1): 52 with RA and 54 with OA, 75.5% of which were women with a median age of 57 years (Interquartile range – IQR 9 years) with no differences between groups. Most of the patients had a low socioeconomic status (54.7%) and the majority had reached secondary school (Supplementary Table 1). Of the total, 8.5% had AH and 1.9%, diabetes mellitus under glycaemic control based on exclusion criteria in both cases. In total 24.1% of OA patients had tendinopathy. Patients with dyslipidemia came to 4.7%, mainly in the OA group (Triglycerides, total cholesterol, and low-density lipoprotein cholesterol were higher), but differences were not significant. Patients with hypothyroidism accounted for 17% (22% were in OA group) and fibromyalgia for 18.9% (37% were in the OA group and differed significantly from the RA group). Almost 50% of patients in the two groups had first-degree relatives with CVD and 28.3% had familial autoimmunity, mainly in the RA group (*p* = 0.023). Patients with OA had significantly higher values for waist and hip circumference, weight, and BMI than RA patients. OA patients did more physical activity than RA patients. Furthermore, there was a significantly higher percentage of pre-obese state and obesity grade I patients in OA group compared to the RA group.

**Table 1:** General variables.

**Variable**		**All subjects *n =* 106 *n* (%)**	**Rheumatoid arthritis *n =* 52 *n* (%)**	**Osteoarthritis *n =* 54 *n* (%)**	***p*-value**
Sex	Female	80 (75.5)	40 (76.9)	40 (74.1)	0.733
SES	Low	58 (54.7)	27 (51.9)	31 (57.4)	0.571
Comorbidities	AH	9 (8.5)	4 (7.7)	5 (9.3)	1.000^a^
	Diabetes mellitus	2 (1.9)	2 (3.8)	0 (0.0)	0.238^a^
	Osteoporosis	12 (11.3)	8 (15.4)	4 (7.4)	0.195
	Dyslipidemia	5 (4.7)	0 (0.0)	5 (9.3)	0.057^a^
	Hypothyroidism	18 (17)	6 (11.5)	12 (22.2)	0.143
	Osteoarthritis	81 (76.4)	27 (51.9)	54 (100)	0.000
	Fibromyalgia	20 (18.9)	0 (0.0)	20 (37)	0.000
Familial CVD - FDR with CVD		51 (48.1)	26 (50)	25 (46.3)	0.703
Familial autoimmunity		30 (28.3)	20 (38.5)	10 (18.5)	0.023
Physical activity		67 (63.2)	31 (59.6)	36 (66.7)	0.452
Lifestyle exposure	Tobacco^b^	8 (7.5)	3 (5.8)	5 (9.3)	0.716^a^
	Coffee	90 (84.9)	47 (90.4)	43 (79.6)	0.122
	Alcohol	4 (3.8)	3 (5.8)	1 (1.9)	0.358^a^
**Variable**		**All subjects** ^**c**^	**Rheumatoid Arthritis** ^**c**^	**Osteoarthritis** ^**c**^	***p*** **-value**
Age (years)		57 (9)	57 (9)	57 (9)	0.934
Waist circumference (cm)		85 (10.5)	82 (10.3)	88.5 (10.3)	0.000
Hip circumference (cm)		101 (9)	98.5 (7)	102 (9.3)	0.002
Waist-hip ratio		0.856 (0.1)	0.848 (0.1)	0.875 (0.1)	0.124
Weight (kg)^d^		65.05 (9.48)	61.78 (9.60)	68.20 (8.3)	0.000
Height (cm)^d^		159.48 (8.46)	158.57 (7.89)	160.35 (8.96)	0.283
BMI (kg/m^2^)^d^		25.9 (5)	24.74 (4)	27.12 (5)	0.000^e^
Fasting plasma glucose (mg/dL)		84 (13)	82.5 (13)	84 (15)	0.173
Triglycerides		1.57 (0.99)^f^ 139 (88)^g^	1.50 (1.02)^f^ 133 (90.3)^g^	1.61 (0.99)^f^ 142.5 (88)^g^	0.808
Total cholesterol^d^		6.12 (1.26)^f^ 237 (48.54)^g^	6.15 (1.17)^f^ 237.98 (45.43)^g^	6.10 (1.34)^f^ 236.07(51.76)^g^	0.410^h^
HDL-c		1.26 (0.53)^f^ 49 (20.5)^g^	1.29 (0.63)^f^ 50 (24.3)^g^	1.22 (0.53)^f^ 47.5 (20.3)^g^	0.726
LDL-c^d^		4.01 (1.02)^f^ 155.21 (39.36)^g^	4.00 (0.95)^f^ 155.01 (36.62)^g^	4.01(1.09)^f^ 155.40 (42.18)^g^	0.480^h^
CRP (mg/dL)		0.400 (0.4)	0.450 (1.2)	0.300 (0.2)	0.003
ESR (mg/dL)		15 (12)	18 (14)	14 (7.5)	0.004
Pain VAS		6 (4)	4.5 (4.8)	7 (2.3)	0.000
VAS GLOBAL		3.5 (3)	3 (3.8)	4 (4)	0.037
Fatigue VAS		3 (6)	2 (4.4)	5 (6.6)	0.008
RAPID3^d^		11.72 (6.29)	10.18 (6.67)	13.21 (5.57)	0.013^h^
VAS Global EQ5D		62.50 (30)	70 (37.5)	57.50 (40)	0.023
EQ-5D-3L VAS score		0.73 (0.22)	0.73 (0.18)	0.69 (0.22)	0.043
EQ-5D-3L TTO score		0.71 (0.33)	0.78 (0.46)	0.64 (0.56)	0.020

a *Fisher test was used;*

b *Smoking cessation based on reference (82);*

c *Median (Interquartile range);*

d *Average (Standard deviation);*

e *U-Mann-Whitney test;*

f *mmo/L;*

g *mg/dL;*

h *t-Student test; AH, Arterial Hypertension; BMI, Body mass index; CRP, C-reactive protein; CVD, Cardiovascular Disease; EQ-5D-3L, European Quality of Life 5 Dimensions 3 Levels; EQ-5D-3L TTO score, European Quality of Life 5 Dimensions 3 Levels time trade-off score; EQ-5D-3L VAS score, European Quality of Life 5 Dimensions 3 Levels visual analog rating scale score; ESR, Erythrocyte sedimentation rate; FDR with CVD, First degree relative with Cardiovascular disease; HDL-c, High-density lipoprotein cholesterol; LDL-c, Low-density lipoprotein cholesterol; RAPID 3, Routine assessment of patient index data 3; SES, Socioeconomic Status; VAS Global EQ5D, Visual analog scale Global European Quality 5 Dimensions; VAS, Visual analog scale*.

Furthermore, close to one third of the patients in each group had received at least one dose of COVID-19 vaccination.

### Clinical and Laboratory Characteristics

Regarding clinical characteristics as measured through the respective scores and scales, OA patients had significantly more pain, fatigue, poor quality of life, and disability compared to RA patients (Table 1). In addition, RA patients had significant higher levels of CRP and ESR when compared to OA patients while the rest of the laboratory measurements showed no differences.

Also, more than 90% of OA patients were under pharmacological treatment, 55.6% of patients were using opioid analgesics, 25.9% currently use neuropathic pain medicine, 20.4% colchicine, and only 3.7% were using non-steroidal anti-inflammatory drugs. Of the total, 40.7% were under physical and rehabilitation follow-up treatment (Table 2 and Supplementary Table 2). There were 79.6% (*n* = 43) OA patients who had generalized polyarticular multisite compromise. Of these, 11 had axial and peripheral compromise.

**Table 2:** Rheumatoid arthritis and osteoarthritis variables.

**Rheumatoid arthritis**		
**Variable**		**Median (IQR)**
Age at onset		41.5 (19)
Age at diagnosis		42.5 (20)
Number of painful joints		2 (6)
Number of swollen joints		2 (2)
DAS-28 (ESR)		3.445 (1.2)
DAS-28 (CRP)		2.610 (1.3)
**Variable**		***n*** **(%)**
Extra-articular manifestations		8 (15.4)
Erosivity		30 (57.7)
RF-positive		44 (84.6)
ACPA-positive		45 (86.5)
Antinuclear antibodies-positive		42 (80.8)
Polyautoimmunity		6 (11.5)
Current treatment	Methotrexate	34 (65.4)
	Other csDMARDs^a^	30 (57.7)
	Antimalarials^b^	3 (5.8)
	GCs	39 (75)
	bDMARDs^c^	17 (32.7)
	Tofacitinib	2 (3.8)
	NSAIDs	5 (9.6)
Previous treatment	csDMARDs	22 (42.3)
	bDMARDs	7 (13.5)
**Osteoarthritis**		
**Variable**		***n*** **(%)**
Patients with Fibromyalgia		20 (37)
Patients with Tendinopathy/Bursitis		13 (24.1)
Inflammatory component of Osteoarthritis		6 (11.1)
Physical and Rehabilitation follow-up treatment		22 (40.7)
Physiotherapy		22 (40.7)
Current treatment	Under pharmacology therapy	51 (94.4)
	NSAIDs	2 (3.7)
	Analgesics, Opioids	30 (55.6)

a *Leflunomide, sulfasalazine;*

b *Chloroquine, Hydroxychloroquine;*

c *abatacept, rituximab, tocilizumab; ACPA, anti-citrullinated peptide antibodies; bDMARDS, biological disease-modifying antirheumatic drugs; CRP, C-reactive protein; csDMARDS, conventional synthetic disease-modifying antirheumatic drugs; ESR, Erythrocyte; GCs, Glucocorticoids; NSAIDs, non-steroidal anti-inflammatory drugs*.

RA patients (Table 2) had low disease activity (DAS-28-CRP), and more than 80% were seropositive (for both RF or ACPA). The median duration of disease was 13.5 years (IQR 14). In the last 3 years, patients have maintained DAS28 values at levels of remission and low activity of the disease (data not shown). Moreover, 11.5% of RA patients had polyautoimmunity, 57.7% were erosive, and 15.4% had extra articular manifestations. Also, 51.9% had both RA and OA. Regarding treatment, 65.4% were under methotrexate (MTX) treatment, 75% were receiving glucocorticoids, and nearly one third were under biological disease-modifying antirheumatic drugs (bDMARDS). Other baseline characteristics of the overall group and specific disease group (including exercise activity level and toxic exposure, etc.) are presented in Table 2 and Supplementary Tables 1, 2.

### Vascular Measurements and Vascular Age Comparisons

There were 35 patients (35.8%) with a VA reported to be greater than 60 years by Arteriograph® measurement. Therefore, the VA was imputed following the proposed methodology. The proportion of the imputed data was similar in the two groups. The median VA in the entire group was 47.50 years (IQR 34) with no significant differences between the two groups. There was a significant difference between the biological age and VA (*p* < 0.01). In addition, the median PWV in the entire group was 8.45 m/s (IQR 3.0) with no significant differences between groups either. The same applied for the aortic augmentation index with a mean of 40.26% (IQR 14.13). Furthermore, there were no significant differences in the other eight cardiovascular variables measured using the Arteriograph TensioMed® on both groups (Table 3).

**Table 3:** Cardiovascular variables.

**Variable**	**All subjects** ***n =* 106 Mean (SD)**	**Rheumatoid Arthritis** ***n =* 52 Mean (SD)**	**Osteoarthritis** ***n =* 54 Mean (SD)**	***p*-value**
Vascular age (years)^a^	47.50 (34)	47.50 (35)	48 (35)	0.635^b^
PWV (m/s)^a^	8.45 (3.0)	8.45 (3.1)	8.45 (2.9)	0.622^b^
Brachial diastolic blood pressure (mmHg)	76.2 (10.54)	77.98 (10.76)	74.51 (10.14)	0.091
Brachial systolic blood pressure (mmHg)	122.98 (14.99)	123.11 (17.33)	122.85 (12.51)	0.928
Brachial pulse pressure (mmHg)^a^	46.50 (12.3)	44 (15.8)	47.5 (9.3)	0.178^b^
Mean arterial pressure (mmHg)	91.64 (11.04)	92.75 (11.52)	90.57 (10.56)	0.313
Heart rate	66.13 (9.49)	66.21 (9.99)	66.05 (9.08)	0.933
Brachial augmentation index (%)	5.45 (28.18)	9.5 (23.75)	1.54 (31.61)	0.147
Central systolic blood pressure (mmHg)^a^	127.35 (21.7)	126.20 (22.3)	128.05 (25)	0.899^b^
Central aortic pulse pressure (mmHg)	49.87 (11.37)	50.10 (12.11)	49.65 (10.72)	0.839
Aortic augmentation index (%)	40.26 (14.13)	42.17 (11.72)	38.41 (16)	0.172
Framingham risk score adjusted for Colombia^a^	9.75 (2.25)	9.75 (1.5)^c^	9.75 (3)	0.926^b^

a *Median (Interquartile range);*

b *U-Mann-Whitney; PWV, Pulse wave velocity; SD, Standard deviation. This was calculated based on the Framingham risk score adjusted for Colombia*.

c *Result for RA patients adapted by a 1.5 multiplication factor following EULAR recommendations was 14.6 (IQR 2.25)*.

Finally, the median percentage of the FRS was 9.7% (IQR 2.2), and there were no differences between groups.

In the total group, those patients who had hypothyroidism had a significantly higher VA compared to those who did not have that comorbidity. Similarly, those patients who were physically active had a lower VA compared to those who did not, but there was only a trend with no statistical significance (Table 4).

**Table 4:** Vascular age values based on different factors.

**Variable**	**All subjects** ***n*** **=** **106**			**Rheumatoid Arthritis** ***n*** **=** **52**			**Osteoarthritis** ***n*** **=** **54**		
**Comorbidities and exercise**	**Presence^**a**^**	**Absence^**a**^**	***p*-value^**b**^**	**Presence^**a**^**	**Absence^**a**^**	***p*-value^**b**^**	**Presence^**a**^**	**Absence^**a**^**	***p*-value^**b**^**
Hypothyroidism	65.99 (52)	46 (34)	0.019	77.59 (63)	46.50 (33)	0.169	65.99 (41)	43 (37)	0.058
Physical Activity	43 (32)	57.7 (36)	0.055	42 (34)	50 (35)	0.179	43 (32)	60.37 (40)	0.177
**Current treatment in rheumatoid arthritis patients**									
**Variable**	**Presence** ^**a**^	**Absence** ^**a**^	***p*** **-value** ^**b**^	-	-	-			
Current treatment with methotrexate	44.5 (21)	70.8 (56)	0.013	-	-	-			
Current treatment with other csDMARDs	58.91 (43)	39.5 (23)	0.002	-	-	-			
Current treatment with glucocorticoids	46 (23)	83.43 (57)	0.054	-	-	-			
Previous treatment with bDMARDs	77.51 (48)	45 (32)	0.021						
**Current management in osteoarthritis patients**									
Follow-up by Physical and Rehabilitation medicine	43 (24)	56.5 (39)	0,018	-	-	-			

a *Median (Interquartile range);*

b *U mann Withney; bDMARDS, biological disease-modifying antirheumatic drugs; csDMARDS, conventional synthetic disease-modifying antirheumatic drugs*.

Doing a subgroup analysis shows that patients within the RA group who received MTX had a lower VA than those who did not receive it (*p* = 0.013). The same occurred for those receiving glucocorticoids although this was only a trend with no statistical significance. Conversely, the patients who received other conventional synthetic disease-modifying antirheumatic drugs (csDMARDs) had a higher VA than those who did not receive them (the majority of the latter, i.e., 81.8% *p* = 0.033, received MTX instead). Finally, those patients who received bDMARDs in the past had a significantly greater VA than those who did not (Table 4).

Within the OA group, in turn, patients who received Physical and Rehabilitation follow-up treatment had lower VA than those who did not (Table 4).

When analyzing VA correlations (Table 5), age correlated with VA in the entire group and in the subgroups. The number of cups of coffee correlated inversely with VA in the entire group. LDL-c levels and the FRS correlated directly with VA in the entire group and in the OA group. The MD-HAQ value also correlated with VA in the RA group. All the correlations were weak or very weak. The only exception was for age which, in turn, was moderate. There were no significant correlations between VA and inflammaging components, nor in the group as a whole or the subgroups.

**Table 5:** Correlations between vascular age and clinical and laboratory variables.

	**All subjects** ***n*** **=** **106**		**Rheumatoid Arthritis** ***n*** **=** **52**		**Osteoarthritis** ***n*** **=** **54**	
**Variable**	**Spearman's rho coefficient**	***p*-value**	**Spearman's rho coefficient**	***p*-value**	**Spearman's rho coefficient**	***p*-value**
Age	0.437	0.000	0.392	0.004	0.492	0.000
Number of cups of coffee	−0.212	0.044	−0.166	0.266	−0.214	0.169
LDL-c	0.213	0.029	0.114	0.42	0.299	0.028
Framingham risk score adjusted for Colombia	0.354	0.000	0.233^a^	0.096	0.457	0.001
MD-HAQ	0.103	0.294	0.276	0.048	−0.023	0.866

*LDL-c, Low-density lipoprotein cholesterol; MD-HAQ, Multidimensional- health assessment questionaire*.

a *The rho coefficient was the same when adapted by a 1.5 multiplication factor following EULAR recommendations*.

There were 68 patients with normal VA and SUPERNOVA, 35 (51.5%) in the OA group and 33 (48.5%) in the RA group, but no significant differences between them were seen. When analyzing patients with EVA, (Supplementary Table 3), 89.5% were women and 10.5% were men (*p* = 0.012). EVA was significantly associated with hypothyroidism, osteoporosis, and FBM (*p* < 0.05). Also, patients with EVA had higher FRS (10.12% IQR 3 vs. 9.7% IQR 2.25, *p* = 0.005). In the RA subgroup, SUPERNOVA was associated with MTX (*p* = 0.001) and glucocorticoid treatment (*p* = 0.031). In contrast, EVA was associated with RA patients receiving treatment with DMARDs other than MTX. Also, RA patients with EVA had higher IL-6 levels (10.12% IQR 3 vs. 9.7% IQR 2.25, *p* = 0.017) when compared to SUPERNOVA patients.

On the other hand, in OA group, there were more patients with SUPERNOVA in the subgroup of patients receiving physical and rehabilitation follow-up treatment when compared to those not receiving it (*p* = 0.006). In addition, patients with OA and fibromyalgia had EVA more frequently than those without fibromyalgia (*p* = 0.019). Lastly, in OA patients, those with EVA had consumed coffee for a longer period compared to those with SUPERNOVA (*p* = 0.05). Detailed results can be seen in Supplementary Table 3.

### Inflammaging

The levels of all the molecules studied in inflammaging panel were higher in the RA group than in the OA group (Table 6) except for MMP-9, TIMP-1 ng/ml, and TIMP-2 ng/ml. These were higher in the OA group, but none of the differences were statistically significant. The RA group simply had a trend toward a higher value of MMP-1 (*p* = 0.05).

**Table 6:** Inflammaging variables.

**Inflammaging variables**	**All subjects *n =* 106 Median (IQR)**	**Rheumatoid arthritis *n =* 52 Median (IQR)**	**Osteoarthritis *n =* 54 Median (IQR)**	***p*- value**
INF-γ pg/ml (*n =* 102)	5.75 (13.23)	5.75 (13.42)	5.47 (13.22)	0.976
IL-10 pg/ml (*n =* 105)	4.01 (4.42)	4.07 (4.69)	3.90 (4.48)	0.992
IL-1RA pg/ml	24.72 (22.53)	27.54 (27.35)	22.29 (18.12)	0.100
IL-1 β pg/ml (*n =* 94)	1.35 (2.37)	1.71 (2.90)	1.10 (1.93)	0.061
IL-6 pg/ml (*n =* 93)	4.58 (14.41)	4.65 (19.61)	4.18 (13.71)	0.320
TNF-α pg/ml	16.12 (11.71)	17.48 (12.41)	14.94 (10.80)	0.499
MMP-1 ng/ml	8.43 (11.19)	10.02 (9.71)	6.72 (12.95)	0.050
MMP-2 ng/ml^a^	203.12 (50.42)	204.62 (49.76)	199.12 (51.47)	0.764^b^
MMP-9 ng/ml	242.06 (236.02)	240.25 (279.88)	244.43 (252.93)	0.250
TIMP-1 ng/ml^a^	77.47 (22.46)	74.09 (25.06)	80.71 (19.33)	0.130^b^
TIMP-2 ng/ml^a^	47.12 (13.33)	45.39 (14.14)	48.79 (12.41)	0.190^b^

a *Average (Standard deviation);*

b *t – Student; IL, Interleukins; IL-1RA, Interleukin - receptor antagonist 1; INF-γ, Interferon-gamma; IQR, Interquartile range; MMP, Metalloproteinases; TIMP, Tissue inhibitors of metalloproteinases; TNF-α, Tumor necrosis factor-alpha*.

### Lipid Profile and Clinical and Laboratory Associations

When analyzing correlations between inflammaging components and lipid profile (Table 7), LDL-c levels correlated with MMP1, TIMP1, and TIMP2 in the entire group. MMP1 also correlated with LDL-c in the RA group, and TIMP1 correlated with LDL-c levels in the OA group. Furthermore, TIMP1 also correlated with cholesterol levels in the OA group. All the correlations were weak or very weak based on the classification of the correlation coefficient (81).

**Table 7:** Significant correlations between lipid profile and inflammaging biomarkers.

	**LDL-cholesterol**						**Total cholesterol**					
**Variable**	**All subjects** ***n*** **=** **106**		**RA** ***n*** **=** **52**		**OA** ***n*** **=** **54**		**All subjects** ***n*** **=** **106**		**RA** ***n*** **=** **52**		**OA** ***n*** **=** **54**	
**Inflammaging**	***r^**a**^***	***p*-value**	***r^**a**^***	***p*-value**	***r^**a**^***	***p*-value**	***r^**a**^***	***p*-value**	***r^**a**^***	***p*-value**	***r^**a**^***	***p*-value**
MMP1	0.231	0.017	0.360	0.009	0.089	0.521	0.192	0.049	0.251	0.072	0.135	0.332
TIMP1	0.200	0.040	0.155	0.271	0.269	0.049	0.236	0.015	0.132	0.351	0.337	0.013
TIMP2	0.194	0.046	0.183	0.193	0.207	0.134	0.240	0.013	0.294	0.035	0.164	0.235

a *Spearman's rho coefficient; MMP, Metalloproteinases; OA, Osteoarthritis; RA, Rheumatoid Arthritis; TIMP, Tissue inhibitors of metalloproteinases*.

## Discussion

To our knowledge, this is the first study to report VA in individuals with RA compared to individuals with OA in a Colombian population. Note that when patients with RA whose disease activity level is very low and under strict control as well as without cardiovascular risk factors are compared to individuals with OA with poor metabolic control and little clinical follow-up, their VA is similar. The following paragraphs discuss the influence of some characteristics on VA in both the total group and the two subgroups.

With advancing age, structural and functional changes in the arterial wall contribute to what has been called vascular aging, and in some prematurely affected subjects even EVA (83). Measurement of arterial stiffness by carotid-femoral PWV represents the best proxy to measure vascular age since an exact VA calculator based on PWV is not available yet (84). In the present analysis, as was expected, age correlated with VA when the total group was analyzed. But it was interesting that there was a significant group of patients, almost two thirds, that could fulfill the SUPERNOVA denominated group given that their VA was lower or the same as biological age. This could possibly be due to the strict exclusion criteria that, apart from some exceptions, prevented the included patients from having higher traditional CVD risks as well as the absence of previous CVD events.

It is well known that patients with RA have an increased CVD risk given the high inflammatory load that affects endothelial function. However, there are few studies of VA in RA patients with low levels of activity or in remission and with few CVD risk factors. Ferraz-Amaro et al. (85) showed that VA is significantly higher than chronological age in patients with RA. These results contrast with ours given that, in our study, 63% (*n* = 33) of the patients with RA had a VA lower than the biological age. It should be noted that in the study by Ferraz-Amaro et al. (85), VA was calculated based on the Systematic Coronary Risk Evaluation (SCORE) algorithm and based on carotid intima media thickness (cIMT). In addition, RA patients in their study had moderate disease activity as shown by the mean DAS-28 while our patients had low disease activity. In their study, there were also a higher proportion of patients with AH, obesity, dyslipidemia, and smoking. All the above and the sum of factors that favor greater VA in RA due to disease activity as well as the traditional CVD risk factors could explain the differences between the two studies. Other authors (20) have shown that smoking, previous cardiovascular events, and high BP had additional significant effects on the vascular aging process. In our study, the number of patients with previous cigarette exposure was low, as expected by the exclusion criteria, and patients with previous cardiovascular events or uncontrolled AH were excluded.

Furthermore, Taverner et al. (86) showed that age, SBP, and body BMI were significantly associated with PWV and collectively accounted for 32% of PWV variability in RA patients. They observed no associations between arterial stiffness and inflammatory variables, disease activity, and duration any more than we did when analyzing VA based on PWV. They concluded, in their population of RA patients that age was the most important variable in determining the increase in PWV. These results contrast with those shown by Wang et al. (87) who, through a metanalysis that included 38 studies, demonstrated that, compared to controls, RA patients had significantly higher levels of PWVcf and meta-regression and that subgroup analysis demonstrated significant association of PWVcf with age, disease duration, and ESR in RA patients.

On the other hand, OA patients have traditionally been considered to have a lower systemic inflammatory component than RA given that, not being an autoimmune disease, its inflammatory load is lower. However, cardiovascular risk has also been described as elevated for these patients, and this has been associated with different factors.

In a study including 3,049 patients with OA, Perrucio et al. (88) found that one third of OA participants were in the highest CVD risk category as measured using a latent CVD risk variable. They concluded OA was associated with worse CVD risk quartiles compared to non-OA. Worse CVD risk quartiles were found among females with multisite OA. BMI was the largest contributor for both sexes. CRP was predominant in females, and metabolic factors and smoking in males. They concluded that their results suggested a potentially important role for inflammation in OA over and above traditional CVD risk factors. In our study, more than two thirds of the OA patients had multisite compromise, and there were no relevant differences between them and RA patients when analyzing cardiovascular measurements. In addition, OA patients have poor metabolic control as shown by higher BMI (with a high proportion of pre-obese state and obesity grade I patients) and higher values of waist and hip circumference than RA patients. These findings could explain, in part, the absence of differences between the two groups in terms of VA in the present study. Moreover, other studies had demonstrated that people with OA had higher augmented central pressure (measured using the Sphygmocor apparatus Atcor®) when compared to controls (89). Correspondingly, Veronese et al. (90) demonstrated that higher values of a multidimensional prognostic index including information on physical function, nutrition, mood, comorbidities, medications, quality of life, and co-habitation status was associated with an increased risk of CVD. In our study, OA patients had a poor quality of life and disability compared to RA patients in addition to their polypharmacy treatment and had multiple comorbidities including fibromyalgia. Interestingly, OA patients who received physical and rehabilitation treatment had lower VA than those who did not. This finding could be explained by a greater motivation to be physically active and make lifestyle changes when they are under this type of treatment, which, in turn, has been shown (91) to have an impact on lower cardiovascular risk in OA patients.

An additional interesting finding in the present study was that patients (RA and OA) with hypothyroidism had higher values of VA compared to those without it. The association between hypothyroidism and increased vascular resistance, arterial wall thickening, and vascular dysfunction is well recognized. In fact Dagre et al. (92) demonstrated that hypothyroidism, even in the subclinical stage, is associated with changes in arterial stiffness when measured by radial artery applanation tonometry. Other authors have also demonstrated that patients with subclinical hypothyroidism may also have endothelial dysfunction (93). In fact, when patients with RA also have hypothyroidism, they present intima-media thickness and show an association with soluble endothelial adhesion molecules apart from risk factors for CVD (94). Also, Sara et al. demonstrated that hypothyroidism in women is associated with microvascular endothelial dysfunction even after adjusting for confounders, and may explain some of the increased risk of CVD in these patients (95). Even though murine studies have demonstrated that hypothyroidism appears to contribute differentially to aging-induced changes in the myocardium and aorta tissues (96). In our study, there was no association with hypothyroidism when the specific subgroups (RA or OA) were analyzed, possibly due to the limited sample size.

Regarding the subgroup of RA patients, note that they were under strict control treatment using the T2T strategy. Therefore, the measurements reflecting endothelial dysfunction, arterial stiffness, and VA were similar to those in OA patients. This would reflect, in some way, a lower CVD risk when considering RA disease activity as a non-traditional cardiovascular risk factor. In this respect, Ruscitti et al. (97) showed, in a study that included 841 RA participants, mostly female, that the achievement and the maintenance of remission reduced the risk of subclinical (*p* = 0.001) and clinical atherosclerosis (*p* = 0.041) defined as the presence of carotid and/or peripheral artery atherosclerotic lesions detected by ultrasound imaging. Additionally, Amigues et al. (98), demonstrated that subclinical myocardial inflammation [(Measured Using 18-Fluorodeoxyglucose Positron Emission Tomography-Computed Tomography (FDG PET-CT)] is frequent in RA. It is associated with RA disease activity and may decrease with RA therapy. Moreover, Tam et al. (99) evaluated 120 patients with early RA randomized to receive 1 year of strict control treatment using the T2T strategy. They found that achieving sustained DAS-28 remission was associated with a significantly greater improvement in PWV. It should also be noted that, in the present study, MD-HAQ levels in RA patients correlated with VA. This takes into consideration the fact that MD-HAQ apart from being useful for measuring the physical function or disability has a clinical value for disease activity assessment in patients with RA. Note that this correlation was very weak.

Furthermore, in the present study, RA patients under MTX treatment had lower VA than those not receiving this treatment. They also had SUPERNOVA when compared to those patients not receiving MTX. MTX has been recognized as a medication that could lower CVD risk in RA patients. Using metanalysis, Micha et al. (100) analyzed the effects of MTX on CVD risk in RA patients and in patients with other articular inflammatory rheumatological conditions. They showed that MTX was associated with a 21% lower risk of total CVD (*n* = 10 studies, 95% CI = 0.73–0.87, *p* < 0.001), and an 18% lower risk of myocardial infarction (*n* = 5, 95% CI = 0.71–0.96, *p* = 0.01). Choi et al. (101) demonstrated that in RA patients MTX may reduce cardiovascular mortality. The MTX mechanism of action is based on the antagonism of purines. The main beneficial action of MTX on cardiovascular system is the reduction of systemic inflammation since, with the decrease in disease activity, cardiovascular risk decreases in the same proportion. In addition, with the use of MTX, oxidative stress is reduced, and this plays a fundamental role in the pathogenesis of systemic inflammatory diseases. It reduces systemic inflammation and oxidative stress and reduces the major CVD risk factors (102). MTX lowers CVD risk in RA, psoriasis, and psoriatic arthritis, but the mechanisms involved have only been partially identified. Data are controversial regarding its effects on endothelial function and atherosclerosis (102).

Moreover, in the present study we found an increased VA in those RA patients receiving csDMARDS other than MTX compared to those not receiving csDMARDs. These results are in line with those shown by Woodman et al. (103) in a study done on 59 RA patients treated with either MTX or other DMARDs. They showed that higher arterial stiffness (measured with PWV at baseline and at 8 months follow-up) preceded increases in BP in subjects with RA treated with csDMARDs. However, this did not occur among those treated with MTX. Nonetheless, there are no studies evaluating the effect of MTX on VA in RA patients.

When RA pharmacological treatments and VA associations are analyzed, patients with previous bDMARDs exposure were found to have higher VA than those not exposed previously. This could be contradictory given that bDMARDs have been shown to reduce the CVD risk in RA patients, especially anti-TNF therapy, which exhibits a variety of myocardial protective mechanisms including promoting cholesterol transport, improving glucose metabolism, downregulating adhesion molecules, and resisting the effects of inflammation on blood coagulation (104). However, there is controversy in terms of their effects on cardiovascular involvement in RA, especially in relation to their effects on lipid profiles (particularly for the IL-6 inhibitors) (105). Furthermore, Trang et al. (106) showed that the inflammation activity at the ascending aorta (measured through positron emission tomography/computed tomography) in 64 RA patients did not change significantly after 6 months of biological treatment. In addition, Bernelot et al. (107) demonstrated that there are activated monocytes and increased inflammation in the arterial wall in a subset of patients with RA in clinical remission despite the use of potent TNF blocking therapies. That is why some researchers have called bDMARDs in RA a double-edged sword in terms of CVD risk management (105). Although the literature is controversial in this regard, no differences were found in VA in the group of patients currently receiving biological therapy when compared to those who did not receive it. It is possible that the finding of higher VA in the group of patients previously exposed to biologicals is related to the chronic burden of the disease. This may have led to a need for biological treatment, and therefore, this association could be considered confounding by indication and something to be explored in the future with a larger sample size.

The only component of the inflammaging panel that was associated with VA in RA patients was IL-6. In this regard, IL-6 levels were higher in those patients with EVA when compared to SUPERNOVA. IL-6 as a mediator of inflammation has been associated with atherosclerosis in RA patients. Also, it has been demonstrated that IL-6 levels affect the lipid profile and result in increased TG and lipolysis of adipose tissue (108, 109). In fact, Rho et al. (110) demonstrated that no matter what the FRS is, IL-6 levels are significantly associated with the severity of subclinical atherosclerosis in 169 RA patients. Also, Batún-Garrido (108) et al. showed that, at higher FRS, there were higher values of leptin and IL-6 in their study population consisting of 77 RA patients.

Regarding the exposure variables, in turn, the results found regarding coffee exposure were contradictory. On the one hand, there was an inverse correlation (weak correlation) between the number of cups of coffee and VA. On the other hand, those patients with EVA had a shorter exposure time to coffee although the latter was borderline significant. In fact, its impact on the cardiovascular system is controversial. Acute caffeine ingestion has been associated with improvement of endothelial function assessed by brachial artery flow mediated dilation in the general population with and without CVD (111). Also, a meta-analysis provided quantitative evidence that consumption of coffee was inversely associated with a dose-response risk of AH (112). In addition, coffee consumption was inversely associated with arterial stiffness in 540 men studied in Japan (113). A strong inverse association between coffee and vascular-related mortality among Hispanics only was also demonstrated in a multiethnic study (114). It should be clarified that neither the type of coffee to which patients were exposed, regular or decaffeinated, nor data regarding tea consumption, nor diet were strictly evaluated. More studies are needed in relation to coffee consumption and CVD risk in RA patients.

Increases in MMPs in atherosclerotic lesions have been described as being involved in the migration of smooth muscle cells into the intima, the rupture of plaques, and the progression of atherosclerosis (115). Furthermore, there are various suggested mechanisms underlying the production of MMP-1 induced by oxidized LDL (oxLDL) that play a pivotal role in atherogenesis (115). This is important for/due to the positive correlations found between LDL levels and MMP1, TIMP1, and TIMP2 in our study. Arai et al. (116) demonstrated that all TIMPs and MMP1 bind to LDL receptor-related protein 1 (LRP1), a multifaceted endocytic and signaling receptor that is responsible for internalization and lysosomal degradation of lipoproteins along with numerous other proteins. They also showed the MMP-1/TIMP-1 complex bound more strongly to LRP1 than either component did alone. This revealed that LRP1 prefers protease-inhibitor complex as a ligand. This is important since MMP-1 degrades collagen and is commonly upregulated in states of disease and injury. Therefore, MMP-1 plays a crucial role in regulating tissue homeostasis, and unregulated or excessive MMP-1 activity can cause extensive tissue damage (116). Moreover, in the present study, MMP1 levels correlated with LDL-c in the RA group. Regarding this correlation, Maldonado et al. (117) demonstrated through *in vitro* analysis that LDL containing immune complexes (that are frequently produced in autoimmune diseases) stimulated MMP1 expression.

Furthermore, Ishikawa et al. (118) demonstrated that incubation of human RA fibroblast-like synoviocytes with ox-LDL increased the dose-dependent production of MMP-1 and MMP-3 proteins. This is significant given that blocking some LDL receptors such as lectin-like ox-LDL receptor 1 [(LOX-1) - the major receptor of ox-LDL] may be a potent therapeutic target for RA at both the local and systemic level which includes the cardiovascular response.

This study has several weaknesses including the lack of precision in the calculation of VA through Arteriograph® for patients with a VA greater than 60. Therefore, there was a need to carry out the imputation process in those cases. However, in order to guarantee the power of the study, given the estimated sample size, multiple imputation was done, which makes a better estimate than a simple imputation. The results were consistent between the set of data without imputing and with the values of the imputation. Moreover, some researchers have argued that VA calculation based on PWV is not accurate. However, there is controversy since other authors think that, in contrast to FRS and SCORE, the PWVcf method is not only a risk calculator but also an exact measurement, which could be an advantage (84). Another weakness of the study was that we did not include another control group that did not have RA nor OA. Given that the study was done during the COVID-19 pandemic, some of the patients received a vaccination while in the inclusion process. However, as already mentioned, there was no difference in the proportions of vaccinated patients between the two groups. In addition, the time between the vaccination and when the samples were taken for the inflammaging analysis was greater than 1 month. Furthermore, the exclusion criteria were strict, because they influence PWV outcomes, but the comorbidities that were excluded were frequent conditions that could be present in patients with RA. Dyslipidemia was not considered an exclusion criterion. There were five cases in the OA group with this clinical antecedent, but at the time of measurement, there was a higher percentage of patients with dyslipidemia in both groups. Additionally, this group of patients presented a higher body mass index and waist-hip ratio than the RA group due to the fact that they do not belong to a strict clinical follow-up program and the increase in sedentary lifestyle related to the COVID-19 pandemic. Another shortcoming was that the subgroup of patients with RA was not large enough to do robust analysis and compare the effects of different current or previous biological therapies and their influence on inflammaging or VA. Likewise, as previously mentioned, a high percentage of this subgroup of patients also had concomitant OA. In OA patients, some variables were not taken into account since they did not have a strict control of the disease. Therefore, the individual progression of the disease could not be evaluated and some laboratory variables could not be measured. Furthermore, the median age of patients in this study was 57+/– 9 years, and this may have led to bias as younger individuals were not included. Finally, in addition to the fact that no differences were found between the VA in the two groups, the correlations found with VA were weak. Therefore, doing a linear regression was not considered prudent.

In conclusion, RA is a well-known chronic, inflammatory, and multi-systemic disease that can increase the CVD risk for the patients who have it. However, when these patients are under a very strict follow-up treatment with a T2T strategy, the activity levels of the disease and the chronic inflammation decrease. Thus, they are likely to have an influence on the CVD risk. If these measurements done on the Colombian population can be validated, they will give us a solid foundation for extrapolating them to other Latin American countries since these populations share similar ethnic varieties and racial admixtures that strongly correspond to the mestizo population which in general have poor RA outcomes (6).

In the present study, when RA patients under strict follow-up and with low levels of disease activity are compared to OA patients who were under non-strict follow up, had poor metabolic control, and low levels of physical function, it may be suggested that they could have a similar cardiovascular risk. However, it is important to highlight that there are many other variables that could influence this absence of differences such as: individual disease progression, response to articular degeneration, cytokines, and prostaglandins production. There were no differences in the VA nor in the inflammaging markers. Furthermore, methotrexate also influences VA in RA patients. This shows that implemented therapies may have an impact on not only the inflammatory state of the joint but also CVD risk. Further studies are recommended to assess CVD in RA patients being treated under a strict T2T strategy.

## Data Availability Statement

The original contributions presented in the study are included in the article/Supplementary Material, further inquiries can be directed to the corresponding author.

## Ethics Statement

The studies involving human participants were reviewed and approved by Ethic Committee for Research on Human Beings (CEISH) by Hospital de San José HSJ-FUCS (Identifier 0173-2019). The patients/participants provided their written informed consent to participate in this study.

## Author Contributions

AR-V, PS-M, P-KB-N, FS-M, and DE, contributed to the concept and design of the study. J-AR-R, G-SR-V, and LS collected and organized the clinical database. AR-V and FS-M did the statistical analysis. DE and LS did the cardiovascular measurements. P-KB-N and L-DG-C coordinated and organized laboratory analysis. SN and AA contributed to the laboratory test. AR-V, G-SR-V, PS-M, P-KB-N, J-AR-R, FS-M, L-DG-C, and DE wrote the original draft. All authors contributed to manuscript revision, read, and approved the submitted version.

## Funding

This work was supported by the Ministry of Science (Minciencias) of Colombia under grant 844-2019 and Fundación Universitaria de Ciencias de la Salud-FUCS contract 829-2019 along with Biomab IPS, Fundación Cardiovascular de Colombia-FCV, La Cardio-Fundación Cardioinfantil financed this study, project code 500784467051.

## Conflict of Interest

The authors declare that the research was conducted in the absence of any commercial or financial relationships that could be construed as a potential conflict of interest.

## Publisher's Note

All claims expressed in this article are solely those of the authors and do not necessarily represent those of their affiliated organizations, or those of the publisher, the editors and the reviewers. Any product that may be evaluated in this article, or claim that may be made by its manufacturer, is not guaranteed or endorsed by the publisher.
