# Supplementary material for: Vascular Age, Metabolic Panel, Cardiovascular Risk and Inflammaging in Patients With Rheumatoid Arthritis Compared With Patients With Osteoarthritis
Source: Front Cardiovasc Med. 2022 Jul 5;9:894577. doi: 10.3389/fcvm.2022.894577 (PMC9295407; doi:10.3389/fcvm.2022.894577)
Supplement: Supplementary file 1 [file Table_1.docx]

Supplementary Material

# Supplementary table 1

| **Variable** | | **All subjects n= 106**  **n (%)** | **Rheumatoid Arthritis n=52**  **n (%)** | **Osteoarthritis n=54**  **n (%)** | ***p*-value**  **(CST)** |
| --- | --- | --- | --- | --- | --- |
| Sex | Male | 26 (24.5) | 12 (23.1) | 14 (25.9) | 0.733 |
| Socioeconomic status | Middle or high | 48 (45.3) | 25 (48.1) | 23 (42.6) | 0.571 |
| Place of birth | -Bogotá  -Cundinamarca (different from Bogota)  -Pacific region  -Atlantic region  -Andean region (different from Bogota and Cundinamarca) | 46 (43.4)  19 (17.9)  1 (0.9)  5 (4.7)  35 (33) | 24 (46.2)  7 (13.5)  0 (0.0)  3 (5.8)  18 (34.6) | 22 (40.7)  12 (22.2)  1 (1.9)  2 (3.7)  17 (31.5) | 0.628 |
| Residence | -Bogota  -Outside Bogota | 95 (89.6)  11 (10.4) | 47 (90.4)  5 (9.6) | 48 (88.9)  6 (11.1) | 0.801 |
| Housing in urban and rural | -Urban  -Rural | 99 (93.4)  7 (6.6) | 48 (92.3)  4 (7.7) | 51 (94.4)  3 (5.6) | 0.713 ^a^ |
| Occupational status | -Intellectual/ office work  -Manual work  -Household duties  -Student  -Dismissed  -Pensionary  -Own business | 5 (4.7)  30 (28.3)  28 (26.4)  1 (0.9)  1 (0.9)  29 (27.4)  12 (11.3) | 3 (5.8)  16 (30.8)  13 (25)  0 (0.0)  0 (0.0)  14 (26.9)  6 (11.5) | 2 (3.7)  14 (25.9)  15 (27.8)  1 (1.9)  1 (1.9)  15 (27.8)  6 (11.1) | 0.871 |
| Educational level | -Primary school  -Secondary school  -Technician  -University  -Postgraduate | 27 (25.5)  42 (39.6)  23 (21.7)  12 (11.3)  2 (1.9) | 12 (23.1)  23 (44.2)  12 (23.1)  4 (7.7)  1 (1.9) | 15 (27.8)  19 (35.2)  11 (20.4)  8 (14.8)  1 (1.9) | 0.726 |
| Marital status | -Single  -Married  -Divorced  -Unmarried cohabitation  -Widowed | 19 (17.9)  50 (47.2)  13 (12.3)  20 (18.9)  4 (3.8) | 10 (19.2)  18 (34.6)  11 (21.2)  11 (21.2)  2 (3.8) | 9 (16.7)  32 (59.3)  2 (3.7)  9 (16.7)  2 (3.7) | 0.035 |
| COVID – 19 vaccine | Get vaccinated against COVID-19 (At least one dose)  -Yes  -No | 34 (32.1)  72 (67.9) | 12 (23.1)  40 (76.9) | 22 (40.7)  32 (59.3) | 0.051 |
|  | Number of doses received  1  2 | 6 (17.6)  28 (82.4) | 2 (16.7)  10 (83.3) | 4 (18.2)  18 (81.8) | 1.000 ^a^ |
|  | ¿Which vaccine did you receive?  - Pfizer  - Sinovac  - AstraZeneca  - Janssen | 23 (67.6)  7 (20.6)  3 (8.8)  1 (2.9) | 7 (58.3)  4 (33.3)  1 (8.3)  0 (0.0) | 16 (72.7)  3 (13.6)  2 (9.1)  1 (4.5) | 0.522 |
| Comorbidities | Kidney disease  Pulmonary diseases  Dermatology diseases  Gastrointestinal diseases  Psiquiatric diseases  Neurologic diseases  Ophthalmology  diseases  Cancer  - Thyroid cancer  - Breast cancer  Previous infectious history  -Septic arthritis  -Covid-19 infection  -Hepatitis B infection  -Latent tuberculosis  Others  Other chronic diseases | 1 (0.9)  8 (7.5)  1 (0.9)  6 (5.7)  3 (2.8)  4 (3.8)  2 (1.9)  3 (2.8)  2 (66.7)  1 (33.3)  4 (3.8)  1 (25)  1 (25)  1 (25)  1 (25)  26 (24.5)  2 (1.9) | 0 (0.0)  1 (1.9)  1 (1.9)  2 (3.8)  1 (1.9)  1 (1.9)  2 (3.8)  1 (1.9)  0 (0.0)  1 (100)  4 (7.7)  1 (25)  1 (25)  1 (25)  1 (25)  11 (21.2)  1 (1.9) | 1 (1.9)  7 (13)  0 (0.0)  4 (7.4)  2 (3.7)  3 (5.6)  0 (0.0)  2 (3.7)  2 (100)  0 (0.0)  0 (0.0)  0 (0.0)  0 (0.0)  0 (0.0)  0 (0.0)  15 (27.8)  1 (1.9) | 1.000 ^a^  0.060 ^a^  0.491 ^a^  0.679 ^a^  1.000 ^a^  0.618 ^a^  0.238 ^a^  1.000 ^a^  0.333 ^a^  0.054 ^a^  0.428  1.000 ^a^ |
| Cardiovascular family history | Relationship  - Mother  - Father  - Brother  What cardiovascular disease was diagnosed?  -Coronary artery disease  - Cerebrovascular disease  Other first degree relative with cerebrovascular disease  Other first degree relative with cardiovascular disease | 21 (41.2)  26 (51)  4 (7.8)  32 (30.2)  16 (15.1)  4 (3.8)  9 (8.5) | 9 (34.6)  16 (61.5)  1 (3.8)  20 (38.5)  4 (7.7)  3 (5.8)  4 (7.7) | 12 (48)  10 (40)  3 (12)  12 (22.2)  12 (22.2)  1 (1.9)  5 (9.3) | 0.247  0.069  0.037  0.358 ^a^  1.000 ^a^ |
| Familial Autoimmunity | First degree relative ^d^  Second degree relative ^e^  One more relative with autoimmune diseases?  What autoimmunity disease was diagnosed?  -Systemic Lupus erythematosus  - Sjögren Syndrome  - Systemic vasculitis  - Rheumatoid arthritis  - Other autoimmune disease | 18 (17)  14 (13.2)  8 (26.7)  9 (8.5)  1 (0.9)  1 (0.9)  18 (17)  1 (0.9) | 10 (19.2)  11 (21.2)  6 (30)  7 (13.5)  0 (0.0)  1 (0.9)  12 (23.1)  0 (0.0) | 8 (14.8)  3 (5.6)  2 (20)  2 (3.7)  1 (0.9)  0 (0.0)  6 (11.1)  1 (1.9) | 0.545  0.018  0.682 ^a^  0.090 ^a^  1.000 ^a^  0.491 ^a^  0.101  1.000 ^a^ |
| Exercise history | Which kind of exercise did you do?  -Aerobic  -Anaerobic  - Both  Exercise frequency  -Daily  -Weekly | 63 (94)  1 (1.5)  3 (4.5)  31 (46.3)  36 (53.7) | 30 (96.8)  0 (0.0)  1 (3.2)  14 (45.2)  17 (54.8) | 33 (91.7)  1 (2.8)  2 (5.6)  17 (47.2)  19 (52.8) | 0.574  0.866 |
| Toxics | Energy drinks  Psychoactive substance  Organic solvents  Biopolymers  Tattoo  Dental amalgam  Hair dyes | 1 (0.9)  1 (0.9)  7 (6.6)  1 (0.9)  13 (12.3)  67 (63.2)  61 (57.5) | 1 (1.9)  1 (1.9)  3 (5.8)  1 (1.9)  5 (9.6)  30 (57.7)  29 (55.8) | 0 (0.0)  0 (0.0)  4 (7.4)  0 (0.0)  8 (14.8)  37 (68.5)  32 (59.3) | 0.491 ^a^  0.491 ^a^  1.000 ^a^  0.491 ^a^  0.415  0.248  0.716 |
| Body mass index (kg/m^2^) | Normal  Pre-obese state  Obesity grade I | 43 (40.6)  54 (50.9)  9 (8.5) | 27 (51.9)  24 (46.2)  1 (1.9) | 16 (29.6)  30 (55.6)  8 (14.8) | 0.012 |
| **Variable** | | **All subjects n= 106**  **Median (IQR)** | **Rheumatoid Arthritis n=52**  **Median (IQR)** | **Osteoarthritis n=54**  **n (%)**  **Median (IQR)** | ***p*-value (MUT)** |
| Smoking Pack-years | | 2 (5.5) | 2 (8.7) | 2 (3) | 0.568 |
| Number of coffee cups  Years of exposure to coffee  Days of coffee consumption | | 2 (2)  35 (10)  7 (2) | 2 (2)  37 (15)  7 (2) | 2 (2)  30 (10)  7 (2) | 0.760  0.151  0.936 |
| Glycosylated hemoglobin A1c % (n=8) | | 5.8 (0.9) | 5.8 (1.1) | 5.8 (0.0) | 1.000 |
| Thyroid-stimulating hormone (n=19) (UI/ml) | | 2.7 (3) | 4.28 (29.7) | 2.03 (2.8) | 0.045 |
| Serum creatinine MR ^c^ (n=98) mg/dL | | 0.79 (0.15) | 0.75 (0.14) | 0.84 (0.14) | 0.631 ^b^ |
| Hemoglobin (n=95) g/dl | | 15 (1.4) | 14.7 (1.1) | 15 (1.8) | 0.236 |
| Leukocytes (n=96) | | 6135 (2110) | 6275  (2425) | 6025  (2028) | 0.055 |
| Platelet | | 274000 | 274000 | 277500 | 0.577 |
| Alanine aminotransferase(n=93) U/L | | 25 (16.8) | 23 (11) | 31 (20) | 0.014 |
| Alkaline phosphatase (n=89) U/L | | 97 (48) | 98 (65.3) | 94 (36.9) | 0.451 |
| Exercise duration in minutes | | 35 (30) | 35 (30) | 37.5 (41.3) | 0.415 |
| **Variables** | | **All subjects n=106**  **Median (IQR)** | **AR n=52**  **Median (IQR)** | **OA n=54**  **Median (IQR)** | ***p- value* (MUT)** |
| Physician VAS | | 4 (4) | 3 (3) | 4 (3.3) | 0.099 |
| Stiffness duration in minutes (n=27) | | 30 (40) | 30 (38.8) | 40 (50) | 0.904 |
| MD-HAQ total | | 6 (10) | 4 (9.0) | 6.5 (9) | 0.236 |
| MD-HAQ Conversion | | 2 (3.3) | 1.3 (3) | 2.15 (3) | 0.236 |
| **Variable** | | **All subjects n=106**  **n (%)** | **AR n=52**  **n (%)** | **OA n=54**  **n (%)** | ***p*-value**  **(CST)** |
| Stiffness in the last week | | 27 (25.5) | 16 (30.8) | 11 (20.4) | 0.219 |
| Get a good night’s sleep?  **-** without any difficulty  - with some difficulty  - with much difficulty  - unable to do | | 30 (28.3)  42 (39.6)  24 (22.6)  10 (9.4) | 21 (40.4)  21 (40.4)  5 (9.6)  5 (9.6) | 9 (16.7)  21 (38.9)  19 (352)  5 (9.3) | 0.005 |
| Deal with feelings of anxiety or being nervous?  **-** without any difficulty  - with some difficulty  - with much difficulty  - unable to do | | 46 (43.4)  48 (45.3)  7 (6.6)  5 (4.7) | 31 (59.6)  17 (32.7)  2 (3.8)  2 (3.8) | 15 (27.8)  31 (57.4)  5 (9.3)  3 (5.6) | 0.011 |
| Deal with feelings of depression or feeling blue?  **-** without any difficulty  - with some difficulty  - with much difficulty  - unable to do | | 47 (44.3)  44 (41.5)  11 (10.4)  4 (3.8) | 29 (55.8)  18 (34.6)  4 (7.7)  1 (1.9) | 18 (33.3)  26 (48.1)  7 (13)  3 (5.6) | 0.121 |

^a^ Fisher test was used; ^b^ t-Student test; ^c^ Average (Standard deviation); ^d^ Parents, children, siblings; ^e^ Uncles, nephew, grandparents, grandchildren and cousins. CST: Pearson's chi-squared test; IQR: Interquartile range; MD-HAQ: Multidimensional health assessment questionnaire; MR: Medical record; MUT: Mann-Whitney U test; VAS: Visual analogue scale.

# Supplementary table 2

| **RA** | | |
| --- | --- | --- |
| **Variable** | | **Median (IQR)** |
| Rheumatoid factor titers (U/ml) | | 139 (236.6) |
| Anti-citrullinated protein antibody titers (U/ml) | | 166.5 (323.7) |
| **Variable** | | **n (%)** |
| Antinuclear antibodies | Antinuclear antibodies dilutions  160  320  640  1280  2560  5120 | (n=41)  7 (17.1)  6 (14.6)  8 (19.5)  13 (31.7)  5 (12.2)  2 (4.9) |
|  | Antinuclear antibodies Hep-2 cell patterns (n40)  Homogeneous  Speckled | 15 (37.5)  25 (62.5) |
| Polyautoimmunity | Sjögren syndrome  Vasculitis | 5 (9.6)  1 (1.9) |
| Medication at baseline | bDMARDs  - Tumor necrosis factor inhibitors  - Others ^b^ | 10 (58.8)  7 (41.2) |
| Previous treatment | Previous use of NSAIDs | 3 (5.8) |
|  | Previous use of GCs | 4 (7.7) |
|  | Previous use of tsDMARDs | 0 (0.0) |
| **OA** | | |
| **Variable** | **n (%)** |  |
| Articular compromise | Generalized polyarticular  Axial and peripheral | 43 (79.6)  11 (25.5) |
| Current treatment | Chondroprotectors | 4 (7.4) |
|  | Diacerein | 7 (13) |
|  | Topical lidocaine | 13 (24.1) |
|  | Antimalarials ^a^ | 4 (7.4) |
|  | Neuropathic pain  medicines ^c^ | 14 (25.9) |
|  | Colchicine | 11 (20.4) |
|  | Without treatment | 3 (5.6) |

^a^Chloroquine, Hydroxychloroquine; ^b^ abatacept, rituximab, tocilizumab; ^c^ Amitriptyline, Duloxetine, Pregabalin, Trazodone; bDMARDS: biological disease-modifying antirheumatic drugs; CRP: C-reactive protein; csDMARDS: conventional synthetic disease-modifying antirheumatic drugs; ESR: Erythrocyte sedimentation rate; GCs: glucocorticoids; IQR: Interquartile range; NSAIDs: non-steroidal anti-inflammatory drugs; tsDMARDS: targeted synthetic disease-modifying antirheumatic drugs.

# Supplementary table 3

**Early vascular age and Supernormal vascular aging in patients with Rheumatoid Arthritis and Osteoarthritis.**

| **Variable in both groups** | | | n (%) |
| --- | --- | --- | --- |
| EVA | | | 38 (35.8) |
| SUPERNOVA | | | 68 (64.2) |
| **Variable** | **RA** n (%) | **OA** n (%) | ***p-*value** ^a^ |
| EVA | 19 (50) | 19 (50) | 0.885 |
| SUPERNOVA | 33 (48.5) | 35 (51.5) |  |
| **Analysis in the whole group** | | | |
| **Variable** | **EVA** n (%) | **SUPERNOVA** n (%) | ***p-*value** ^a^ |
| Sex  Male  Female | 4 (10.5)  34 (89.5) | 22 (32.4)  46(67.6) | 0.012 |
| AH  Presence  Absence | 6 (15.8)  32 (84.2) | 3(4.4)  65 (95.6) | 0.067 ^b^ |
| Osteoporosis  Presence  Absence | 8 (21.1)  30 (78.9) | 4 (5.9)  64 (94.1) | 0.026 ^b^ |
| Hypotiroidism  Presence  Absence | 11 (28.9)  27 (71.1) | 7 (10.3)  61 (89.7) | 0.014 |
| Fibromyalgia  Presence  Absence | 11 (28.9)  27 (71.1) | 9 (13.2)  59 (86.8) | 0.047 |
| **Variable** | **EVA** | **SUPERNOVA** | ***p****-***value** ^c^ |
| Framingham risk score adjusted for Colombia Median (IQR) | 10.12 (3) | 9.75 (2.25) | 0.005 |
| **Rheumatoid arthritis patients** | | | |
| **Variable** | **EVA** n (%) | **SUPERNOVA** n (%) | ***p-*value** ^a^ |
| Current treatment with methotrexate  Yes  No | 7 (36.8)  12 (63.2) | 27 (81.8)  6 (18.2) | 0.001 |
| Current treatment with other csDMARDs  Yes  No | 15 (78.9)  4 (21.1) | 15 (45.5)  18 (54.5) | 0.019 |
| Current treatment with glucocorticoids  Yes  No | 11 (57.9)  8 (42.1) | 28 (84.8)  5 (15.2) | 0.047 ^b^ |
| Previous treatment with bDMARDs  Yes  No | 5 (26.3)  14 (73.7) | 2 (6.1)  31 (93.9) | 0.085 ^b^ |
| **Variable** | **EVA** | **SUPERNOVA** | ***p****-***value** ^c^ |
| IL-6 pg/ml (n=93) Median (IQR) | 9.24 (35.1) | 3.76 (7.26) | 0.017 |
| **Osteoarthritis patients** | | | |
| **Variable** | **EVA** n (%) | **SUPERNOVA** n (%) | ***p-*value** ^a^ |
| Follow-up by Physical and Rehabilitation medicine  With the follow up  Without the follow up | 3 (15.8)  16 (84.2) | 19 (54.3)  16 (45.7) | 0.006 |
| Fibromyalgia  Presence  Absence | 11 (57.9)  8 (42.1) | 9 (25.7)  26 (74.3) | 0.019 |
| **Variable** | **EVA** | **SUPERNOVA** | ***p****-***value** ^c^ |
| Time of coffee exposure (years) Median (IQR) | 35 (15) | 30 (20) | 0.050 |

^a^ Chi-squared test; ^b^ Fisher test; ^c^ U-Mann-Whitney test; AH: Arterial Hypertension; bDMARDS: biological disease-modifying antirheumatic drugs; csDMARDS: conventional synthetic disease-modifying antirheumatic drugs; EVA: Early vascular aging; IL-6 IL: Interleukin-6; IQR: Interquartile range; SUPERNOVA: Supernormal or normal vascular aging.
